# HBV infection in untreated HIV-infected adults in Maputo, Mozambique

**DOI:** 10.1371/journal.pone.0181836

**Published:** 2017-07-31

**Authors:** Lúcia Mabalane Chambal, Eduardo Samo Gudo, Awa Carimo, Rita Corte Real, Nédio Mabunda, Cremildo Maueia, Adolfo Vubil, Ana Flora Zicai, Nilesh Bhatt, Francisco Antunes

**Affiliations:** 1 Hospital Central de Maputo, Maputo, Mozambique; 2 Instituto Nacional de Saúde, Maputo, Mozambique; 3 Faculdade de Medicina, Universidade Eduardo Mondlane, Maputo, Mozambique; 4 Laboratório de Biologia Molecular–Centro Hospitalar de Lisboa Central, Lisboa, Portugal; 5 Instituto de Saúde Ambiental–Faculdade de Medicina da Universidade de Lisboa, Lisboa, Portugal; University of Cincinnati College of Medicine, UNITED STATES

## Abstract

**Background:**

HIV/ HBV coinfected patients are at high risk of developing chronic HBV infection, liver cirrhosis and hepatocellular carcinoma. In Mozambique, where HIV prevalence is one of the highest in the world, HIV-infected patients are scarcely characterized in terms of HBV coinfection and 3TC-resistance mutations profile.

**Methods:**

To characterize ART-naïve HIV-infected adults, with and without HBV coinfection, a cross-sectional study was conducted between May and November 2012 in two health centers from Maputo city, Mozambique. Subjects were consecutively enrolled in the study and, then, tested for hepatitis B surface antigen (HBsAg). Moreover, CD4^+^ T cells count, HBV DNA in plasma, HBV genotyping and 3TC-resistance mutations profile of HBV were assessed in HIV/HBV coinfected patients.

**Results:**

In total, 518 patients were enrolled in the study. The median age was 33 years old and 66.8% were women. The median CD4^+^ T cells count was 361 cells/mm^3^ and 47 (9.1%) were coinfected with HBV. Out of 46 coinfected patients, 24 (55.2%) had HBV DNA ≥ 20 - < 20 000 and 12 (26.1%) had HBV-DNA ≥20 000. APRI > 2.0 was reported in 4.3% of coinfected and 1.7% of monoinfected patients (p = 0.228), while FIB-4 > 3.25 was reported in 4.4% of coinfected and 1.3% of monoinfected patients (p = 0.112). Genotype A was the most frequent, identified in 25/27 (92.6%) patients, whereas genotype E was present in 2/27 (7.4%) patients. No patient had 3TC-resistance mutations.

**Conclusions:**

This study showed that HBV coinfection was prevalent among ART-naïve HIV-infected adults in Mozambique. Overall, these data highlight the importance of screening HBV coinfection as an integrated measure of HIV routine care to improve health conditions and treatment of HIV/HBV coinfected patients.

## Introduction

Human immunodeficiency virus (HIV) infection is a major public health problem in sub-Saharan Africa, where it is estimated to live 2/3 of the 34 million people globally infected with HIV [1]. In the last decade, coinfection with hepatitis B virus (HBV) became a serious concern among HIV-infected patients, because both viruses share similar routes of infection. In addition, data from Western cohorts show that HIV impacts on almost every aspect of the natural history of HBV infection [2, 3]. Among the worldwide HIV-infected individuals, 5–15% are estimated to be coinfected with HBV [2, 4–6]. Consequences include higher rates of chronicity after acute HBV infection, higher level of HBV replication, more rapid progression of liver disease, higher liver-related mortality, and increased risk of hepatotoxicity to antiretroviral drugs [7–12]. Also in Western cohorts, liver disease has emerged as a leading cause of death in HIV-infected individuals coinfected with either hepatitis B or C [12–15]. Recent studies found that coinfection with HBV can also lead to increased progression in outcomes related to Acquired Immune Deficiency Syndrome (AIDS) and all-cause mortality among HIV-infected patients [16, 17]. Moreover, the burden of coinfection is greater in limited resources regions, such as the sub-Saharan Africa [2, 4–6]. Recently, HIV/HBV coinfection in sub-Saharan Africa has received medical attention, as almost all African countries are scaling up their antiretroviral (ARV) therapy (ART) programs towards the ambitious target of universal coverage and elimination of HIV transmission [18–20]. Despite the World Health Organization (WHO) recommendations to test hepatitis B surface antigen (HBsAg) in all HIV-infected patients [21], this testing is rarely performed in sub-Saharan Africa, due to its unavailability. Additionally, based on WHO guidelines, patients with HIV/HBV coinfection should start ART regimen with at least two active drugs for HBV infection, such as tenofovir (TDF) with emtricitabine (FTC) or lamivudine (3TC). As a result of the rapid growth of ART programs in sub-Saharan Africa and of the unknown status for HBV infection, when the therapy starts, a general concern has been raised about HBV mutations causing 3TC-resistance [22–24]. In spite of this, data regarding the primary resistance of HBV to 3TC in Southern African countries is still insufficient. For instance, Mozambique has the eighth highest HIV prevalence in the world and the prevalence of HIV/HBV coinfection is 13.6% [1, 25–27]. In this country, 3TC and TDF are currently available in the national program for HIV treatment and are recommended in HIV/HBV coinfected patients. However, no national guidelines for screening and treatment of HBV infection are available and liver disease monitoring is not performed routinely, within the national health system.

A recent study conducted in a district of northern Mozambique reported a low rate of resistance to 3TC on HIV/HBV coinfected patients initiating ART; however, data on this matter are not available for the rest of the country. In the southern region of Mozambique, HIV epidemic is worse. Thus, intervention on HBV primary resistance to 3TC among ART naïve HIV-infected patients is needed, since the test-start approach to control this epidemic has been recently launched in the country [28, 29].

Data on HBV genetic diversity is also crucial for disease management, considering that different HBV genotypes might influence the response to antiviral therapy and the clinical outcomes [30–32].

In this context, this study aimed to determine the prevalence, genetic diversity and the profile of HBV primary resistance to 3TC among ART naïve HIV-infected patients, attending outpatient clinics in Maputo city, Mozambique.

## Methods

Between May and November 2012, a cross-sectional study was conducted in two health centers from the suburban area of Maputo city, Mozambique. Both sites provide primary health care services to HIV-infected individuals in outpatient settings. This local belongs to a large suburban area of Maputo city known for its poverty and high population density, associated with precarious housing, low literacy and income (informal labor) and poor sanitation.

This study was approved by the National Bioethics Committee of Mozambique. ART naïve HIV-infected patients aged > 18 years were consecutively enrolled after signing the informed consent form. Participants were recruited from a cohort of pre-ART HIV-infected patients followed at the two sites: the Mavalane Health Center and the Polana Caniço Health Center. Pregnant women were not included in this study. For laboratory analysis, a total of 10mL of whole blood was collected from each participant. Sociodemographic characteristics, clinical and laboratory data were recorded in a standardized form. Patients were stratified according to HBsAg serological results: HIV-infected/HBsAg negative patients (mono-infected) and HIV-infected/HBsAg positive patients (coinfected).

All participants performed a complete blood count (Sysmex KX21N: Sysmex Corporation, Sysmex), serum transaminases assays (ABX pentra 400: Horiba ABX SAS, France) and CD4+ T cells count (BD FACSCalibur, CA, USA). First, patients were screened for HBsAg (Diasorin S.p.A., Sallugia, Italy). The ones with positive HBsAg were measured for HBV DNA viral load with COBAS AmpliPrep/COBAS TaqMan HBV test (version 2.0, Roche Diagnostics, Germany; detectability cut-off: 20 IU/mL), which is a fully-automated system that employs real time PCR technology. Briefly, this system uses a set of primers to amplify a highly-conserved pre-Core/Core region of the HBV genome in all eight genotypes (A-H). The protocol for amplification is pre-programmed into the COBAS AmpliPrep/COBAS TaqMan auto analyzer.

HBV genotyping and assessment of HBV drug resistance mutations to 3TC were performed using TRUGENE HBV Genotyping Kit Module 2.0 and the OpenGene DNA sequencing system (Siemens Healthcare Diagnostics Inc, Tarrytown, USA). This device is a fully automated and integrated system that amplifies a fragment of approximately 1.2 Kb, corresponding to a portion of the surface antigen (S) (s101-s237) and the overlapping reverse transcriptase (RT) gene (rt99-rt280). Sequencing of the amplified products was performed using the CLIP^TM^ sequencing technology (Visible Genetics), as previously described [33]. Detection of the DNA mutations’ profile, responsible for the resistance to 3TC, was based on the phylogenetic analysis of the sequenced fragment. For this purpose, a library of validated and known reference HBV mutants’ sequences (consensus sequences) was used. The analysis was performed using GeneObjects^TM^ and GeneLibrarian^TM^ module of the OpenGene DNA Sequencing System, as previously described [34]. Genotyping was performed in all samples considering HBV DNA ≥ 300 UI/mL.

For liver disease staging, AST (aspartate aminotransferase), ALT (alanine aminotransferase) and platelet count measurements allowed calculation of FIB-4 (Fibrosis 4 index) and APRI (AST-Platelet Ratio Index). These non-invasive tests were assessed using the formula described elsewhere [35]. According to WHO guidelines, an APRI score > 2.0 indicates significant liver fibrosis and cirrhosis, while a FIB-4 score > 3.25 indicates significant cirrhosis [36].

The sample size was calculated using One-sample test for proportions considering a HBV prevalence of 10.6%, a precision of 3% and a confidence interval (CI) level of 95%. Data were double entered in a secure and de-identified database developed using Microsoft Access™ 2007. Analysis was performed using the statistics package STATA 12.0 (StataCorp, College Station, TX, USA). For univariate analysis, the Mann-Whitney U test was used to compare numerical variables and Pearson’s chi-squared test was used to compare categorical variables. Statistical significance was considered when p-value was < 0.05.

## Results

In this study, 518 ART-naïve HIV-infected patients were enrolled. The median age of the study population was 33 years old (interquartile range [IQR]: 28–42 years) and 66.8% were women. HBsAg was reactive in 47 patients, yielding a coinfection rate of 9.1%.

Overall, 4 (<1%) patients reported previous HBV immunization, 56 (10.8%) reported blood transfusion, while 219 (42.3%) and 78 (15.1%) reported ritual scarification and tattoo/piercings, respectively.

Table 1 presents the sociodemographic and clinical results according to a stratification by mono-infected or HIV/HBV coinfected patients. There were no significant differences between the HIV monoinfected group and the HIV/HBV coinfected group in terms of age, sex, sexual behavior, exposure to blood (ritual scarification and tattoo/piercings), HBV vaccination and alcohol consumption. However, history of blood transfusion was higher in monoinfected than in coinfected patients (17.7% *versus* 2.2%; p = 0.047). It is worth noting that both groups reported high-risk behaviors, such as high frequency of unprotected sexual intercourse (36.4% in monoinfected *versus* 48.9% in coinfected patients; p = 0.09), as well as multiple sexual partners (80.9% in monoinfected *versus* 83.0% in coinfected patients; p = 0.723). Ritual scarification was also frequent in both groups (42.9% *versus* 41.3% in mono-infected and coinfected patients, respectively; p = 0.833).

**Table 1:** Comparison of sociodemographic, risk factors and clinical characteristics between mono-infected and coinfected participants.

Characteristics		HIV +	HIV +/HBsAg +	*p-value*	
		(n = 471)	(n = 47)		
**Median age, years (IQR)**	33 (28–42)	32 (28–42)	0.581
**Gender**			
Male (%)	155 (33.2)	15 (33.3)	
Female (%)	312 (66.8)	30 (66.7)	0.944
**Multiple partners**			
Yes (%)	380 (80.9)	39 (83.0)	
No (%)	90 (19.1)	8 (17.0)	0.723
**Unprotected sexual intercourse**			
Yes (%)	171 (36.4)	23 (48.9)	
No (%)	299 (63.6)	24 (51.1)	0.090
**Scarification**			
Yes (%)	200 (42.9)	19 (41.3)	
No (%)	266 (57.1)	27 (58.9)	0.833
**Tattoo/piercings**			
Yes (%)	73 (15.6)	5 (11.4)	
No (%)	395 (84.4)	39 (88.6)	0.455
**Blood transfusion**			
Yes (%)	55 (11.7)	1 (2.2)	
No (%)	415 (88.3)	45 (97.8)	**0.047**
**Prior HBV vaccination**			
Yes (%)	3 (0.6)	1 (2.2)	
No (%)	466 (99.4)	45 (97.8)	0.258
**Alcohol consumption**			
Yes (%)	176 (37.5)	19 (40.4)	
No (%)	294 (62.5)	28 (59.6)	0.688

Furthermore, 2/3 of the patients were classified as HIV stage I and stage II (WHO staging system), with similar distribution among both groups (Table 2). Mono- and coinfected subjects had similar hemoglobin level, leukocytes, lymphocytes, platelets, CD4+T cell counts, and serum transaminase levels. Clinical signs of liver disease (e.g., jaundice, ascites, splenomegaly and hepatomegaly) were not observed in either groups (data not shown).

**Table 2:** Comparison of laboratory characteristics between coinfected and mono-infected patients.

Characteristics	HIV +	HIV+/HBsAg +	p-value
	(n = 471)	(n = 47)	
**WHO Clinical Stage**			
Stage I (%)	210 (45.5)	22 (46.8)	
Stage II (%)	138 (29.9)	14 (29.8)	
Stage III (%)	109 (23.6)	10 (21.3)	
Stage IV (%)	4 (0.9)	1 (2.1)	0.848
**Median hemoglobin, g/dL (IQR)**	11.6 (10.2–13.0)	12.1 (11.0–12.8)	0.288
**Median leucocyte count, 10**^**9**^**/L (IQR)**	4.7 (3.8–5.8)	4.5 (3.6–5.2)	0.177
**Median lymphocyte count, 10**^**9**^**/L (IQR)**	2.0 (1.0–2.0)	2.0 (1.0–2.0)	0.186
**Median platelets count (IQR)**	226 (177–269)	212 (176–286)	0.278
**Median CD4**^**+**^**T–cell count, cells/mm**^**3**^ **(IQR)**	363 (204–508)	327 (131–462)	0.203
**Median ALT, IU (IQR)**	21.4 (16.0–30.1)	26.2 (18.0–35.0)	0.054
**Median AST (IQR)**	28.8 (22.1–38.1)	29.4 (26.0–39.8)	0.244
**Median HBV DNA, IU/mL (IQR)**		1484 (244–727,292)	
**HBV DNA categories (IU/mL)**			
< 20	-	10 (21.7)	
≥ 20 - < 20 000		24 (52.2)	
≥20 000		12 (26.1)	
**Median APRI (IQR)**	0.3 (0.2–0.5)	0.4 (0.3–0.5)	0.117
**APRI scores**			
**< = 2.0**	460 (98.3)	45 (95.7)	
**>2.0**	8 (1.7)	2 (4.3)	0.228
**Median FIB-4 (IQR)**			
**FIB-4 scores**	0.8 (0.6–1.2)	0.9 (0.7–1.3)	0.231
**< = 3.25**	458 (98.7)	44 (95.6)	
**>3.25**	6 (1.3)	2 (4.4)	0.112

APRI—aminotransferase-to-platelet ratio index; DNA–desoxirribonucleic acid; IU–International Units; HBV–hepatitis B virus; HIV–human immunodeficiency virus; FIB-4—fibrosis index based on the four factors; IQR = interquartile range; < 20 IU/mL (undetectable).

Plasma HBV-DNA determination was performed in 46 of the 47 coinfected participants, of whom 10 (21.7%) had undetectable HBV DNA levels (<20 IU/mL). Among samples with detectable levels of HBV, 24 (52.2%) patients had HBV DNA levels between 20 IU/mL and 20 000 IU/mL and 12 (26.1%) patients had ≥ 20 000 IU/mL (Table 2). Results of hepatic fibrosis assessed by APRI and FIB-4 showed that coinfected and monoinfected patients had similar median values of APRI (0.4 coinfected versus 0.3 in monoinfected) and FIB-4 (0.9 in coinfected versus 0.8 in monoinfected). Also, the frequency of patients with APRI score >2 (4.3% in coinfected *versus* 1.7% in monoinfected patients), and FIB-4 score > 3.25 (4.4% in coinfected *versus* 1.3% in monoinfected) was not statistically different.

**Fig 1:**
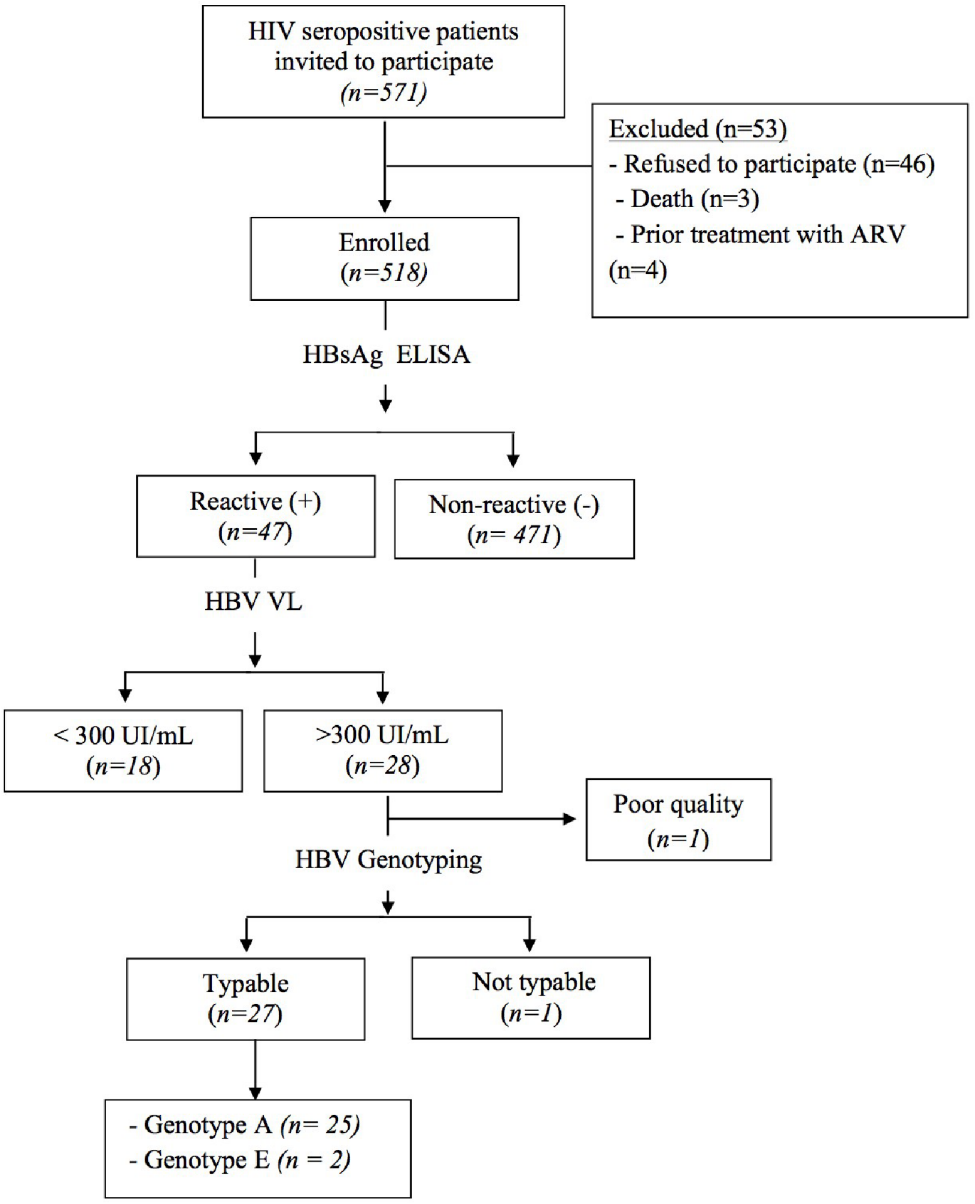
Flowchart of participant’s recruitment and testing. The flow chart depicts the naïve HIV-infected patients enrolled in the study from two health centers in Maputo, between May and November 2012. Abbreviations: ARV–antiretroviral treatment; HBV VL–Hepatitis B viral load.

Genotyping testing for HBV was performed in 27 patients. One sample with HBV-DNA > 300 IU/mL was not typeable due to its poor quality as shown in Fig 1. Genotype A was detected in 25 (92.6%) and genotype E was detected in 2 (7.4%) coinfected patients. No 3TC-resistance associated with HBV mutations were detected in any of the tested samples.

## Discussion

To the best of our knowledge, this represents the first study addressing clinical and laboratory characteristics of HIV/HBV coinfection, HBV DNA viral load, and profile of HBV resistance to 3TC in southern Mozambique. The burden of HIV-infection in this region is the heaviest over the country. The prevalence of coinfection was determined to be 9.1%, which was slightly lower than previously reported in the region [25, 27]. However, the obtained prevalence in southern Mozambique was superior to the values reported in the northern region [28], and in other countries from sub-Saharan Africa [24, 37–39]. Mozambique is estimated to have more than 1.5 million people living with HIV infection [1, 26]. Based on the current literature and in our study, we anticipate that approximately 150,000 people might be coinfected with HIV/ HBV. Thus, intervening in those cases is a major concern, as coinfected patients are at higher risk of developing liver toxicity to ART, cirrhosis, hepatocelular carcinoma (HCC) and death [40, 41]. In this study, we quantified HBV DNA levels in plasma of HIV/HBV coinfected patients, since this parameter is considered a strong predictor of liver-related diseases (e.g., cirrhosis and HCC) [12, 42, 43]. Indeed, our data showed that 26.1% of the coinfected patients had a HBV viral load >20,000 UI/mL. This finding is in line with what was found in another study conducted in developing countries, where a significant proportion of HIV/HBV coinfected patients present HBV DNA≥ 20,000 IU/mL [44].

We also assessed APRI and FIB-4 scores, the two most widely studied non-invasive instruments for assessing liver fibrosis in patients with Hepatitis B and C, in absence of liver biopsy or imaging resources, such as ultrasound or fibroscan [45]. According to our measurement, APRI and FIB-4 scores were not significantly different in coinfected and monoinfected patients. This can be explained by the fact that in this study, most participants were immunocompetent. Indeed, previous studies have shown that coinfected patients are at higher risk of developing liver fibrosis and cirrhosis [46]. As management of HIV/HBV coinfection in sub-Saharan Africa region is hindered by commonly unavailable HBsAg screening and DNA testing in routine care, most HIV infected patients are treated with ART before detection of HBV infection [22, 24]. Therefore, routine HBsAg screening should be implemented in Mozambique and in other countries from this region, where both virus are endemic, to ensure that all coinfected patients are treated with regimens containing TDF plus 3TC or FTC. 3TC is a backbone option of first line ART regimen, globally recommended and recognized as well tolerated and active against both HIV and HBV [47]. Prior studies have shown that coinfected people with lower HBV DNA levels (<20,000 IU/mL) or who are HBeAg-negative, can be considered for 3TC monotherapy, when TDF is not readily available or is contraindicated [44] Briefly, no genetic mutations related to HBV resistance to 3TC were found in the S Ag nor in RT genes. In contrast to our finding, recent studies conducted in northern Mozambique and in other sub-Saharan countries found mutations associated with primary HBV resistance to 3TC [28, 48]. While our results suggest that 3TC resistance in southern Mozambique might be very low, previous studies show that for ARV experienced patients, 3TC-resistance emerged in coinfected patients, after 12–24 months of treatment [39, 49, 50]. Thus, this observation emphasizes that surveillance of HBV resistance profile among ART experienced patients might be critical to improve the national algorithms for HBV treatment.

In this study, genotype A was present in 92.6% of the HBV tested samples. Similar findings were also reported among blood donors in Maputo city during 2004 [51], and among HIV-infected adults in northern Mozambique [28]. Surprisingly, we found that none of the coinfected patients presented signs of decompensated liver disease (e.g., jaundice, ascites, splenomegaly and hepatomegaly). In contrast, other studies have shown that coinfection was associated with higher risk of cirrhosis, HCC, liver decompensation and death, particularly in those with low CD4^+^ T cell counts [40]. This difference might be expected since 75% of the patients included in our study were classified as stage I/II for HIV infection and showed a relatively intact immune system (median CD4+T cell count: 327 cells/mm^3^).

Regarding the impact of HBV on HIV disease progression, previous studies did not find evidence that HBV might accelerate or aggravate the natural history of HIV-infection [52]. Indeed, our data showed that CD4+T cell counts were similar in coinfected and mono-infected patients. However, due to the cross-sectional design of our study, we could not conclude about the impact of HBV on HIV disease progression. To answer this question, well-designed prospective studies are needed.

The present study has some limitations. Firstly, the cross-sectional design did not allow to assess the longitudinal impact of HIV/HBV coinfection in the progression to AIDS. Secondly, because TRUGENE is a fully automated and closed system, we could not draw the phylogenetic tree for HBV sequenced fragments. Lastly, due to the relatively small size of the coinfected group, several differences between coinfected and monoinfected groups might not have reached statistical significance.

## Conclusions

This study strengthens the knowledge on HIV/HBV coinfection in Mozambique and also allowed to characterize this new cohort in Maputo City. We found that one third of HIV/HBV coinfected patients presents high levels of HBV DNA and are at risk of developing a liver-related disease. This finding raises a serious public health concern, which highlights the need to identify HIV/HBV coinfection in these populations. Finally, this study reinforces the importance of integrating HBV screening programs into HIV routine care to reduce morbidity and mortality levels caused by HIV/HBV coinfection.

## Supporting information

S1 File Study database. (XLS)

## References

[1] UNAIDS. AIDSInfo 2015 2015. Available from: http://aidsinfo.unaids.org/.

[2] Hoffmann CJ, Thio CL. Clinical implications of HIV and hepatitis B co-infection in Asia and Africa. Lancet Infect Dis. 2007;7(6):402–9. doi: 10.1016/S1473-3099(07)70135-4 . 17521593 10.1016/S1473-3099(07)70135-4

[3] Puoti M, Torti C, Bruno R, Filice G, Carosi G. Natural history of chronic hepatitis B in co-infected patients. J Hepatol. 2006;44(1 Suppl):S65–70. doi: 10.1016/j.jhep.2005.11.015 . 16338021 10.1016/j.jhep.2005.11.015

[4] Alter MJ. Epidemiology of viral hepatitis and HIV co-infection. J Hepatol. 2006;44(1 Suppl):S6–9. doi: 10.1016/j.jhep.2005.11.004 . 16352363 10.1016/j.jhep.2005.11.004

[5] Easterbrook P, Sands A, Harmanci H. Challenges and priorities in the management of HIV/HBV and HIV/HCV coinfection in resource-limited settings. Semin Liver Dis. 2012;32(2):147–57. doi: 10.1055/s-0032-1316476 . 22760654 10.1055/s-0032-1316476

[6] Modi AA, Feld JJ. Viral hepatitis and HIV in Africa. AIDS Rev. 2007;9(1):25–39. . 17474311

[7] Colin JF, Cazals-Hatem D, Loriot MA, Martinot-Peignoux M, Pham BN, Auperin A, et al Influence of human immunodeficiency virus infection on chronic hepatitis B in homosexual men. Hepatology. 1999;29(4):1306–10. doi: 10.1002/hep.510290447 . 10094979 10.1002/hep.510290447

[8] Laure F, Zagury D, Saimot AG, Gallo RC, Hahn BH, Brechot C. Hepatitis B virus DNA sequences in lymphoid cells from patients with AIDS and AIDS-related complex. Science. 1985;229(4713):561–3. . 2410981 10.1126/science.2410981

[9] Konopnicki D, Mocroft A, de Wit S, Antunes F, Ledergerber B, Katlama C, et al Hepatitis B and HIV: prevalence, AIDS progression, response to highly active antiretroviral therapy and increased mortality in the EuroSIDA cohort. AIDS. 2005;19(6):593–601. . 15802978 10.1097/01.aids.0000163936.99401.fe

[10] Law WP, Dore GJ, Duncombe CJ, Mahanontharit A, Boyd MA, Ruxrungtham K, et al Risk of severe hepatotoxicity associated with antiretroviral therapy in the HIV-NAT Cohort, Thailand, 1996–2001. AIDS. 2003;17(15):2191–9. doi: 10.1097/01.aids.0000076348.42412.3a . 14523276 10.1097/00002030-200310170-00007

[11] Puoti M, Spinetti A, Ghezzi A, Donato F, Zaltron S, Putzolu V, et al Mortality for liver disease in patients with HIV infection: a cohort study. J Acquir Immune Defic Syndr. 2000;24(3):211–7. . 10969344 10.1097/00126334-200007010-00003

[12] Thio CL, Seaberg EC, Skolasky R Jr., Phair J, Visscher B, Munoz A, et al HIV-1, hepatitis B virus, and risk of liver-related mortality in the Multicenter Cohort Study (MACS). Lancet. 2002;360(9349):1921–6. . 12493258 10.1016/s0140-6736(02)11913-1

[13] Weber R, Sabin CA, Friis-Moller N, Reiss P, El-Sadr WM, Kirk O, et al Liver-related deaths in persons infected with the human immunodeficiency virus: the D:A:D study. Arch Intern Med. 2006;166(15):1632–41. doi: 10.1001/archinte.166.15.1632 . 16908797 10.1001/archinte.166.15.1632

[14] Salmon-Ceron D, Lewden C, Morlat P, Bevilacqua S, Jougla E, Bonnet F, et al Liver disease as a major cause of death among HIV infected patients: role of hepatitis C and B viruses and alcohol. J Hepatol. 2005;42(6):799–805. . 15973779 10.1016/j.jhep.2005.01.022

[15] Joshi D, O'Grady J, Dieterich D, Gazzard B, Agarwal K. Increasing burden of liver disease in patients with HIV infection. Lancet. 2011;377(9772):1198–209. doi: 10.1016/S0140-6736(10)62001-6 . 21459211 10.1016/S0140-6736(10)62001-6

[16] Chun HM, Roediger MP, Hullsiek KH, Thio CL, Agan BK, Bradley WP, et al Hepatitis B virus coinfection negatively impacts HIV outcomes in HIV seroconverters. The Journal of infectious diseases. 2012;205(2):185–93. doi: 10.1093/infdis/jir720 ; PubMed Central PMCID: PMCPMC3244364. 22147794 10.1093/infdis/jir720 PMC3244364

[17] Nikolopoulos GK, Paraskevis D, Hatzitheodorou E, Moschidis Z, Sypsa V, Zavitsanos X, et al Impact of hepatitis B virus infection on the progression of AIDS and mortality in HIV-infected individuals: a cohort study and meta-analysis. Clin Infect Dis. 2009;48(12):1763–71. doi: 10.1086/599110 . 19435436 10.1086/599110

[18] United Nations GA, Sixty-fifth session. Political Declaration on HIV/AIDS: Intensifying our Efforts to Eliminate HIV/AIDS2011 April 5th, 2016. Available from: http://www.unaids.org/sites/default/files/sub_landing/files/20110610_UN_A-RES-65-277_en.pdf.

[19] Organization WH. THE GLOBAL HEALTH SECTOR STRATEGY ON HIV/AIDS 2011–2015: AN INTERIM REVIEW OF PROGRESS. 2015.

[20] UNAIDS. 90–90–90—An ambitious treatment target to help end the AIDS epidemic. 2014.

[21] Organization WH. Guidelines for the prevention, care and treatment for persons with chronic hepatitis B infection World Health Organization, 2015 3 2015. Report No. 26225396

[22] Di Bisceglie AM, Maskew M, Schulze D, Reyneke A, McNamara L, Firnhaber C. HIV-HBV coinfection among South African patients receiving antiretroviral therapy. Antivir Ther. 2010;15(3 Pt B):499–503. doi: 10.3851/IMP1494 ; PubMed Central PMCID: PMCPMC3001165. 20516571 10.3851/IMP1494 PMC3001165

[23] Kim HN, Scott J, Cent A, Cook L, Morrow RA, Richardson B, et al HBV lamivudine resistance among hepatitis B and HIV coinfected patients starting lamivudine, stavudine and nevirapine in Kenya. J Viral Hepat. 2011;18(10):e447–52. doi: 10.1111/j.1365-2893.2011.01466.x ; PubMed Central PMCID: PMCPMC3177102. 21914062 10.1111/j.1365-2893.2011.01466.x PMC3177102

[24] Ocama P, Seremba E, Apica B, Opio K. Hepatitis B and HIV co-infection is still treated using lamivudine-only antiretroviral therapy combination in Uganda. Afr Health Sci. 2015;15(2):328–33. doi: 10.4314/ahs.v15i2.4 ; PubMed Central PMCID: PMCPMC4480502. 26124776 10.4314/ahs.v15i2.4 PMC4480502

[25] Naniche D, Letang E, Nhampossa T, David C, Menendez C, Alonso P. Alterations in T cell subsets in human immunodeficiency virus-infected adults with co-infections in southern Mozambique. Am J Trop Med Hyg. 2011;85(4):776–81. doi: 10.4269/ajtmh.2011.10-0713 ; PubMed Central PMCID: PMCPMC3183791. 21976586 10.4269/ajtmh.2011.10-0713 PMC3183791

[26] Saúde INd, Estatística INd, Macro I. Inquérito Nacional de Prevalência, Riscos Comportamentais e Informação sobre o HIV e SIDA em Moçambique—INSIDA 200. Maputo, Mozambique: 2010.

[27] Ramanlal NA. Serological markers of Hepatitis B and C virus among HIV infected and high risk uninfected individuals attending Voluntary Counselling and Testing centre for HIV in Maputo, Mozambique University of Sidney University of Sidney 2008.

[28] Wandeler G, Musukuma K, Zurcher S, Vinikoor MJ, Llenas-Garcia J, Aly MM, et al Hepatitis B Infection, Viral Load and Resistance in HIV-Infected Patients in Mozambique and Zambia. PloS one. 2016;11(3):e0152043 doi: 10.1371/journal.pone.0152043 ; PubMed Central PMCID: PMCPMC4816321. 27032097 10.1371/journal.pone.0152043 PMC4816321

[29] MISAU. Guião de implementação da abordagem do testar e iniciar Maputo: MISAU, 2016.

[30] Aoudjane S, Chaponda M, Gonzalez Del Castillo AA, O'Connor J, Noguera M, Beloukas A, et al Hepatitis B virus sub-genotype A1 infection is characterized by high replication levels and rapid emergence of drug resistance in HIV-positive adults receiving first-line antiretroviral therapy in Malawi. Clin Infect Dis. 2014;59(11):1618–26. doi: 10.1093/cid/ciu630 ; PubMed Central PMCID: PMCPMC4650769. 25100867 10.1093/cid/ciu630 PMC4650769

[31] Cao GW. Clinical relevance and public health significance of hepatitis B virus genomic variations. World J Gastroenterol. 2009;15(46):5761–9. PubMed Central PMCID: PMCPMC2791267. doi: 10.3748/wjg.15.5761 19998495 10.3748/wjg.15.5761 PMC2791267

[32] Sanchez-Tapias JM, Costa J, Mas A, Bruguera M, Rodes J. Influence of hepatitis B virus genotype on the long-term outcome of chronic hepatitis B in western patients. Gastroenterology. 2002;123(6):1848–56. doi: 10.1053/gast.2002.37041 . 12454842 10.1053/gast.2002.37041

[33] Kessler HH, Stelzl E, Marth E, Stauber RE. Detection of mutations in the hepatitis B virus polymerase gene. Clin Chem. 2003;49(6 Pt 1):989–92. . 12766008 10.1373/49.6.989

[34] Germer JJ, Charlton MR, Ishitani MB, Forehand CD, Patel R. Characterization of hepatitis B virus surface antigen and polymerase mutations in liver transplant recipients pre- and post-transplant. Am J Transplant. 2003;3(6):743–53. . 12780567 10.1034/j.1600-6143.2003.00149.x

[35] Lurie Y, Webb M, Cytter-Kuint R, Shteingart S, Lederkremer GZ. Non-invasive diagnosis of liver fibrosis and cirrhosis. World J Gastroenterol. 2015;21(41):11567–83. doi: 10.3748/wjg.v21.i41.11567 ; PubMed Central PMCID: PMCPMC4631961. 26556987 10.3748/wjg.v21.i41.11567 PMC4631961

[36] WHO. Guidelines for the prevention, care and treatment of persons with chronic hepatitis B infection Geneve, Switzerland: WHO, 2015. 26225396

[37] Hamers RL, Zaaijer HL, Wallis CL, Siwale M, Ive P, Botes ME, et al HIV-HBV coinfection in Southern Africa and the effect of lamivudine- versus tenofovir-containing cART on HBV outcomes. J Acquir Immune Defic Syndr. 2013;64(2):174–82. doi: 10.1097/QAI.0b013e3182a60f7d . 23892239 10.1097/QAI.0b013e3182a60f7d

[38] Tremeau-Bravard A, Ogbukagu IC, Ticao CJ, Abubakar JJ. Seroprevalence of hepatitis B and C infection among the HIV-positive population in Abuja, Nigeria. Afr Health Sci. 2012;12(3):312–7. ; PubMed Central PMCID: PMCPMC3557671. 23382745 10.4314/ahs.v12i3.10 PMC3557671

[39] Calisti G, Muhindo R, Boum Y 2nd, Wilson LA, Foster GM, Geretti AM, et al Epidemiology of HBV infection in a cohort of Ugandan HIV-infected patients and rate and pattern of lamivudine-resistant HBV infection in patients receiving antiretroviral therapy. Trans R Soc Trop Med Hyg. 2015;109(11):723–9. doi: 10.1093/trstmh/trv077 . 26386408 10.1093/trstmh/trv077

[40] Burnett RJ, Francois G, Kew MC, Leroux-Roels G, Meheus A, Hoosen AA, et al Hepatitis B virus and human immunodeficiency virus co-infection in sub-Saharan Africa: a call for further investigation. Liver Int. 2005;25(2):201–13. doi: 10.1111/j.1478-3231.2005.01054.x . 15780040 10.1111/j.1478-3231.2005.01054.x

[41] Kumar R, Singla V, Kacharya S. Impact and management of hepatitis B and hepatitis C virus co-infection in HIV patients. Trop Gastroenterol. 2008;29(3):136–47. . 19115605

[42] Mendy ME, Welzel T, Lesi OA, Hainaut P, Hall AJ, Kuniholm MH, et al Hepatitis B viral load and risk for liver cirrhosis and hepatocellular carcinoma in The Gambia, West Africa. Journal of viral hepatitis. 2010;17(2):115–22. doi: 10.1111/j.1365-2893.2009.01168.x ; PubMed Central PMCID: PMCPMC2817443. 19874478 10.1111/j.1365-2893.2009.01168.x PMC2817443

[43] Chen CJ, Yang HI, Su J, Jen CL, You SL, Lu SN, et al Risk of hepatocellular carcinoma across a biological gradient of serum hepatitis B virus DNA level. JAMA. 2006;295(1):65–73. doi: 10.1001/jama.295.1.65 . 16391218 10.1001/jama.295.1.65

[44] Li Y, Xie J, Han Y, Wang H, Zhu T, Wang N, et al Lamivudine Monotherapy-Based cART Is Efficacious for HBV Treatment in HIV/HBV Coinfection When Baseline HBV DNA <20,000 IU/mL. J Acquir Immune Defic Syndr. 2016;72(1):39–45. doi: 10.1097/QAI.0000000000000927 ; PubMed Central PMCID: PMCPMC4977191. 26745828 10.1097/QAI.0000000000000927 PMC4977191

[45] Xiao G, Yang J, Yan L. Comparison of diagnostic accuracy of aspartate aminotransferase to platelet ratio index and fibrosis-4 index for detecting liver fibrosis in adult patients with chronic hepatitis B virus infection: a systemic review and meta-analysis. Hepatology. 2015;61(1):292–302. doi: 10.1002/hep.27382 . 25132233 10.1002/hep.27382

[46] Xie J, Han Y, Qiu Z, Li Y, Li Y, Song X, et al Prevalence of hepatitis B and C viruses in HIV-positive patients in China: a cross-sectional study. J Int AIDS Soc. 2016;19(1):20659 doi: 10.7448/IAS.19.1.20659 ; PubMed Central PMCID: PMCPMC4793284. 26979535 10.7448/IAS.19.1.20659 PMC4793284

[47] Europe WROf. Management of hepatitis B and HIV coinfection—Clinical Protocol for the WHO European Region (2011 revision)2011 March, 25th, 2016. Available from: http://www.euro.who.int/__data/assets/pdf_file/0011/152012/e95792.pdf.

[48] Selabe SG, Lukhwareni A, Song E, Leeuw YG, Burnett RJ, Mphahlele MJ. Mutations associated with lamivudine-resistance in therapy-naive hepatitis B virus (HBV) infected patients with and without HIV co-infection: implications for antiretroviral therapy in HBV and HIV co-infected South African patients. J Med Virol. 2007;79(11):1650–4. doi: 10.1002/jmv.20974 . 17854040 10.1002/jmv.20974

[49] Benhamou Y, Bochet M, Thibault V, Di Martino V, Caumes E, Bricaire F, et al Long-term incidence of hepatitis B virus resistance to lamivudine in human immunodeficiency virus-infected patients. Hepatology. 1999;30(5):1302–6. doi: 10.1002/hep.510300525 . 10534354 10.1002/hep.510300525

[50] Selabe SG, Song E, Burnett RJ, Mphahlele MJ. Frequent detection of hepatitis B virus variants associated with lamivudine resistance in treated South African patients infected chronically with different HBV genotypes. J Med Virol. 2009;81(6):996–1001. doi: 10.1002/jmv.21479 . 19382250 10.1002/jmv.21479

[51] Cunha L, Plouzeau C, Ingrand P, Gudo JP, Ingrand I, Mondlane J, et al Use of replacement blood donors to study the epidemiology of major blood-borne viruses in the general population of Maputo, Mozambique. Journal of medical virology. 2007;79(12):1832–40. doi: 10.1002/jmv.21010 . 17935167 10.1002/jmv.21010

[52] Sinicco A, Raiteri R, Sciandra M, Bertone C, Lingua A, Salassa B, et al Coinfection and superinfection of hepatitis B virus in patients infected with human immunodeficiency virus: no evidence of faster progression to AIDS. Scandinavian journal of infectious diseases. 1997;29(2):111–5. . 9181644 10.3109/00365549709035869

