# Correction: HBV infection in untreated HIV-infected adults in Maputo, Mozambique

**DOI:** 10.1371/journal.pone.0190460

**Published:** 2017-12-21

**Authors:** Lúcia Mabalane Chambal, Eduardo Samo Gudo, Awa Carimo, Rita Corte Real, Nédio Mabunda, Cremildo Maueia, Adolfo Vubil, Ana Flora Zicai, Nilesh Bhatt, Francisco Antunes

There is an error in the second author's name in the citation. The correct citation is: Chambal LM, Gudo ES, Carimo A, Corte Real R, Mabunda N, Maueia C, et al. (2017) HBV infection in untreated HIV-infected adults in Maputo, Mozambique. PLoS ONE 12(7): e0181836. https://doi.org/10.1371/journal.pone.0181836
